# Supplementary material for: Plasmodium ARK2-EB1 axis drives the unconventional spindle dynamics, scaffold formation and chromosome segregation of sexual transmission stages
Source: Res Sq. 2023 Feb 8:rs.3.rs-2539372. Preprint. [Version 1] doi: 10.21203/rs.3.rs-2539372/v1 (PMC9934748; doi:10.21203/rs.3.rs-2539372/v1)
Supplement: 1 [file NIHPPRS2539372V1-supplement-1.pdf]

SUPPLEMENTARY DATA

Supplementary figures

Fig S1. Generation of PbARK2-GFP parasites and analysis of subcellular location of ARK2-GFP throughout the life cycle

(A) Schematic representation of the endogenous *Pbark2* locus, the GFP-tagging construct and the recombined *ark2* locus following single homologous recombination. Arrows 1 and 2 indicate the position of PCR primers used to confirm successful integration of the construct. **(B)** Diagnostic PCR of *ark2* and WT parasites using primers IntT204 (Arrow 1) and ol492 (Arrow 2). Integration of the ark2 tagging construct gives a band of 594 bp. Tag = ARK2-GFP parasite line. **(C)** Live cell imaging of ARK2-GFP parasites during erythrocytic schizogony showing one or two focal points of ARK2-GFP (green) per nucleus. DNA is stained with Hoechst dye (blue); scale bar = 5 µm. **(D)** Live cell imaging of ARK2-GFP parasites during oocyst development in mosquitoes showing discrete foci of ARK2-GFP. DNA is stained with Hoechst dye (blue); scale bar = 5 µm. **(E)** Live cell imaging showing ARK2-GFP gametocytes at 30 sec and 15 min after activation. ARK2-GFP was not detected in free gametes (15 min gametocytes). DNA is stained with Hoechst dye (blue); scale bar = 5 µm. **(F)** Live-cell imaging showing ARK2-GFP location in zygote and ookinete. A cy3-conjugated antibody, 13.1, which recognises the protein P28 on the surface of zygotes and ookinetes was used to mark these stages (red). DNA is stained with Hoechst dye (blue); scale bar = 5 µm. **(G)** Still images (at every 5 s) showing dynamic location of ARK2-GFP in gametocytes within 3 to 4 min post activation (mpa) during male gametogony. DNA is stained with Hoechst dye (blue); scale bar = 5 µm. **(H)** Still images (at every 5 s) showing dynamic location of ARK2-GFP within 6 to 7 mpa during male gametogony. DNA is stained with Hoechst dye (blue); scale bar = 5 µm.

Fig S2. Quantification and staining with tubulin antibody of events of ARK2 localization during male gametogony.

A. The events of ARK2-GFP localization during different time points after gametocytes activation. **(B)** Immunofluorescence assay (IFA) showing location of ARK2 (green) and α-tubulin (red) in male gametocytes at different time points after activation**.** DNA is stained with DAPI (blue); mpa = min post activation; scale bar = 5 µm. **(C)** Deconvoluted images improve the resolution of ARK2 and show its colocalization with spindle microtubules. Scale bar = 5 µm.

Fig S3. The location of ARK2 and various subcellular markers

(A) The location of ARK2-mCherry (red) and the kinetochore marker, NDC80-GFP (green) during male gametogony. DNA is stained with Hoechst dye (blue); scale bar = 5 µm**.** **(B)** Still images (at every 5 s) showing dynamic location of ARK2-mCherry and NDC80-GFP in gametocytes activated for 2 to 3 min. DNA is stained with Hoechst dye (blue); scale bar = 5 µm. **(C)** The location of ARK2-GFP (green) and the basal body and axoneme marker, kinesin-8B-mCherry (red) during male gametogony. DNA is stained with Hoechst dye (blue); scale bar = 5 µm. **(D)** Still images (at every 5 s)

showing dynamic location of ARK2-GFP and kinesin-8B-mCherry in gametocytes activated for 4 to 5 min. DNA is stained with Hoechst dye (blue); scale bar = 5 μm.

Fig S4. ARK2 associates with spindle microtubules.

(A) STED confocal microscopy showing co-localization of ARK2 (purple) and α-tubulin (green) at spindle but not with cytoplasmic microtubules in gametocytes activated for 2 min. DNA is stained with SiR DNA (blue); scale bar = 1 μm. **(B)** Expansion microscopy showing co-localization of ARK2 (yellow) and α/β tubulin (purple) staining at spindle but not at cytoplasmic microtubules at 2 mpa. Scale bar = 1 μm. **(C)** 3D-SIM image showing locations of ARK2 (purple) and NDC80 (green) at 2 mpa. Scale bar = 1 μm. DNA (blue) is stained with DAPI.

Fig S5. Generation and genotypic analysis of *Pb*ARK2-AID/HA and P_{clag}-*ark2* parasites.

(A) Schematic representation of auxin inducible degron (AID) strategy to generate *ARK2-AID/HA* parasites. (**B).** Integration PCR of the ARK2-AID/HA construct in the *ark2* locus. Oligonucleotides used for PCR genotyping are indicated, and agarose gels to analyse the corresponding PCR products from genotyping reactions are shown. (**C)** ARK2-AID/HA protein expression level as measured by western blotting upon addition of auxin to mature purified gametocytes; α-tubulin served as a loading control. **(D)** Male gametogony (Exflagellation rate) of ARK2-AID/HA as measured upon addition of auxin and without auxin to mature purified gametocytes. **(E)** Schematic representation of the promoter swap strategy to construct *Pclag-ark2* parasites (placing ARK2 under the control of the clag promoter) by double homologous recombination. Arrows 1 and 2 indicate the primer positions used to confirm 5' integration and arrows 3 and 4 indicate the primers used to confirm 3' integration **(F)** Integration PCR of the promotor swap construct into the *ARK2* locus. Primer 1 (IntPTD245) and primer 2 (5'-IntPTD) were used to confirm successful integration of the selectable marker, resulting in a band of 460 bp. Primer 3 (3'-intPTclag) and primer 4 (IntPTD243) were used to determine the successful integration of the clag promoter, resulting in a band of 571 bp. Primer 1 (IntPTD245) and primer 4 (IntPTD243) were used to confirm a complete knock-in of the construct with a band at 4.5 kb and the absence of a band at 2.1 kb. **(G)** qRT-PCR showing normalised expression of ARK2 transcripts in P_{clag}-*ark2* and WT-GFP parasites.

Fig S6. Analysis of ookinete motility of P_{clag}-*ark2* and WT-GFP parasites

(A) Representative frames from time-lapse videos of WT-GFP and *Pclag-ark2* ookinetes in matrigel. Red arrow indicates the apical end of the ookinetes. Bar = 5 μm. **(B)** Graph shows the quantitative data for WT-GFP and *Pclag-ark2* ookinete motility. (Error bar ± SD; n=3 independent experiments; >20 ookinetes were analysed for each experiment). **(C)** RNA sequence analysis showing downregulated transcript of ARK2 in *Pclag-ark2* parasites. **(D)** Gene ontology enrichment analysis showing the most affected genes involved in various biological processes.

Fig S7. Generation of PbEB1-GFP parasites and analysis of PbEB1-GFP location during gametogony

(A) Schematic representation of the endogenous *Pbeb1* locus, the GFP-tagging construct and the recombined *eb1* locus following single homologous recombination. Arrows 1 and 2 indicate the position of PCR primers used to confirm successful integration of the construct. **(B)** Diagnostic PCR of *eb1* and WT parasites using primers IntT264 (Arrow 1) and ol492 (Arrow 2). Integration of the EB1 tagging construct gives a band of 1267 bp. Tag = EB1-GFP parasite line. **(C)** Still images (at every 5 s) showing dynamic location of EB1-GFP in activated gametocytes at 1-2 min during male gametogony. DNA is stained with Hoechst dye (blue); scale bar = 5 μm. **(D)** Still images (at every 5 s) showing dynamic location of EB1-GFP in activated gametocytes at 2 to 3 mpa. DNA is stained with Hoechst dye (blue); Scale bar = 5 μm. **(E)** Still images (at every 5 s) showing dynamic location of EB1-GFP in activated gametocytes at 4 to 6 mpa. DNA is stained with Hoechst dye (blue); scale bar = 5 μm. **(F)** The location of EB1-GFP (green) and the kinetochore marker, NDC80-mCherry (red) during male gametogony. DNA is stained with Hoechst dye (blue); scale bar = *5* μm**.**

Fig S8. EB1 associates with spindle microtubules.

(A) 3D-SIM image showing location of EB1 (green) with NDC80 (purple) in gametocyte activated for 2 min and EB1 (green) with ARK2 (purple) in gametocytes activated for 4 min. 3D-SIM images showing location of EB1 (purple) and cytoplasmic SAS4 (green) in gametocyte activated for 2 min. DNA is stained with Hoechst dye (blue); scale bar = 1 μm. (B) STED confocal microscopy showing co-localization of EB1 (purple) and α-tubulin (green) at spindle but not with cytoplasmic microtubules in gametocytes activated for 2 min. DNA is stained with SiR DNA (blue); scale bar = 1 μm.

Fig S9. PbEB1-GFP is located at the apical end of the parasite and at the putative MTOC and spindle like PbARK2-GFP during ookinete development

Live-cell imaging shows that EB1-GFP is located at the microtubule organising centre (MTOC) and spindles in the nucleus during ookinete development and then disappears in mature ookinetes (24 h). It is also located at the apical end of the growing protuberance during zygote to ookinete transition. A cy3-conjugated antibody, 13.1, which recognises the protein P28 on the surface of zygotes and ookinetes was used to mark these stages (red). Scale bar = 5 μm.

Fig S10. Generation and genotypic analysis of *Δeb1* parasites

(A) Schematic representation of the endogenous *eb1* locus, the targeting knockout construct and the recombined *eb1* locus following double homologous crossover recombination. Arrows 1 and 2 indicate PCR primers used to confirm successful integration in the *eb1* locus following recombination, and arrows 3 and 4 indicate PCR primers used to show deletion of the *eb1* gene. (**B)** Integration PCR of the *eb1* locus in WTGFP (WT) and knockout (Mut) parasites using primers: integration primer and ol248. Integration of the targeting construct gives band of expected size for each gene.

(C) Gene ontology enrichment of upregulated genes in global transcriptomic analysis of *Δeb1* gametocytes activated for 30 min, showing where the most affected genes are involved in various biological processes.

Supplementary tables

Table S1. Overview of genomes and sequences used for generating Figure 1B.

Table S2. List of genes differentially expressed between P_{clag}-*ark2* and WT-GFP gametocytes activated for 30 min.

Table S3. Spreadsheet (excel) file with unique peptide values for GFP-trap immunoprecipitate for gametocytes 1 minute after activation for WT-GFP, ARK2-GFP and EB1-GFP parasites. NAs are set to zero (0). Specific protein groups that belong to a similar functional class (e.g. replication machinery, kinetochore etc) are colour coded according to the scheme visualised in Fig 5B and Fig 8. Five parts of the table are present: (1) gene details I; containing gene name, manual annotations, amino acid number (AA) and molecular weight (MW), (2) correlations; Pearson (p) and Spearman (s, rank) correlation values for ARK2 and EB1, (3) PCA, principal components, (4) unique peptide values; NA is -, and * indicates that single peptide calls are to be approached with suspicion (minimal of 2 is usual cut-off), (5) gene details II; for GO terms and OG definitions that can be found at PlasmoDB (https://plasmodb.org/).

Table S4. List of genes differentially expressed between *Δeb1* and WT-GFP gametocytes activated for 30 min

Table S5. Oligonucleotides used in this study.

Supplementary Movies

Video S1. Time lapse video showing ARK2-GFP focal point extending to form a bridge-like spindle and breaking into two halves in gametocytes 1 to 2 min after activation. Still images used in Fig 2B.

Video S2. Time lapse video showing two ARK2-GFP bridge-like spindles breaking and producing four focal points in gametocytes 3 to 4 min after activation. Still images used in Fig S1G.

Video S3. Time lapse video showing four ARK2-GFP bridge-like spindles breaking and producing eight focal points in gametocytes 6 to 8 min after activation. Still images used in Fig S1H.

Video S4. Time lapse video showing ARK2-mCherry and NDC80-GFP dynamics in gametocytes activated for 1 to 2 min. Still images used in Fig 2D.

Video S5. Time lapse video showing ARK2-mCherry and NDC80-GFP dynamics in gametocytes activated for 2 to 3 min. Still images used in Fig S2D.

Video S6. Time lapse video showing ARK2-GFP and kinesin-8B-mCherry dynamics in activated gametocytes for 2-3 min. Still images used in Fig 2F.

Video S7. Time lapse video showing ARK2-GFP and kinesin-8B-mCherry dynamics in gametocytes activated for 4 to 6 min. Still images used in Fig S2F.

Video S8. Gliding motility of *Pclag-ark2* ookinetes. Still images used in Fig S4A

Video S9. Gliding motility of *WT-GFP* ookinetes. Still images used in Fig S4A

Video S10. Time lapse video showing EB1-GFP focal point extending to form a bridge like spindle in activated gametocytes for 1-2 min. Still images used in Fig S5C.

Video S11. Time lapse video showing EB1-GFP bridge breaking into two halves and accumulating at two focal points in a gametocyte activated for 2 to 3 min. Still images used in Fig S5D.

Video S12. Time lapse video showing two bridges of EB1-GFP breaking into four halves and accumulating at four focal points in a gametocyte activated for 2 to 3 min. Still images used in Fig S5E.
